# Evaluating Nurses’ Perceptions of Documentation in the Electronic Health Record: Multimethod Analysis

**DOI:** 10.2196/69651

**Published:** 2025-04-28

**Authors:** Deborah Jacques, John Will, Denise Dauterman, Kathleen Evanovich Zavotsky, Barbara Delmore, Glenn Robert Doty, Kerry O'Brien, Lisa Groom

**Affiliations:** 1 Department of Health Informatics, Medical Center Information Technology, NYU Langone Health, One Park Avenue, 12 Fl., New York, NY, 10016, United States, 1 646-784-1726; 2 Department of Nursing, Center for Innovations in the Advancement of Care, NYU Langone Health, New York, NY, United States; 3 Department of Population Health, Grossman School of Medicine, New York University, New York, NY, United States

**Keywords:** electronic health record, nurse, documentation burden, focus group, usability, documentation

## Abstract

**Background:**

Nurses are one of the largest user groups of the electronic health record (EHR) system, relying on its tools to support patient care and nursing workflows. Recent studies suggested that the redesign of nursing documentation may reduce the time spent in the EHR system and improve nurse satisfaction.

**Objective:**

We aimed to assess nurses’ perceptions of the redesigned EHR, evaluate the impact of documentation interventions, and identify future improvement needs.

**Methods:**

Guided by the American Nursing Informatics Association’s Six Domains of Burden conceptual framework, this multimethod project combined both qualitative and quantitative approaches. Registered nurses across the academic health system were recruited via email invitations to participate in focus group discussions. The focus groups were conducted via a web conference and ranged from 60 to 90 minutes in duration. The focus group discussions were transcribed and analyzed through thematic analysis. The EHR vendor’s time data were used to analyze nurses’ time spent in documentation.

**Results:**

In total, 20 registered nurses participated in the focus group discussions, and 17 nurses completed the demographic survey; 88% (15/17) of participants had ≥3 years of EHR experience at the academic health system, and 53% (9/17) self-reported being competent in the EHR system. The following six themes emerged: positive feedback, usability and workflow opportunities, nuisance, training and education, communication, and time spent in the system. EHR vendor time data revealed that the time spent in flowsheets averaged 31.11% per 12-hour shift.

**Conclusions:**

Overall, participants reported a positive experience and that the EHR supported patient care. There are opportunities to further reduce redundancies in documentation and implement programs that support continuous learning about EHR and health technology tools. Specific suggestions include optimizing the oral health assessment tool. Analyzing frontline nursing perspectives in the redesign of EHR workflows is imperative for identifying interventions that support nurses’ satisfaction with the EHR.

## Introduction

### Background

Nursing documentation is critical for high-quality patient care and effective communication among health care professionals. Before the implementation of electronic health records (EHRs), clinician documentation was primarily recorded by using paper-based methods [1]. The Health Information Technology for Economic and Clinical Health (HITECH) Act, which passed in 2009 in the United States, aimed to improve health care quality and safety and encourage the efficient use of health ITs, such as the EHR [2]. Hospitals were incentivized to implement EHR systems, resulting in 98.3% of hospitals adopting electronic-based systems in recent decades [3]. The increased sophistication of EHR systems has introduced documentation requirements and clinician decision support tools, potentially increasing clinicians’ documentation burden [4]. The American Medical Informatics Association (AMIA) describes *documentation burden* as the stress resulting from excessive work that is required to document in the EHR [5].

Nurses are one of the largest user groups of the EHR system and are primary users of flowsheet tools for documentation [6]. Flowsheets are structured tools in the EHR system; they are used to record discrete data over time and are designed in a tabular format. Resembling a spreadsheet, each column represents a date and time, while each row is designed to capture selectable options or free-text values. Nurses capture assessments and observations in flowsheets and, on average, document 631 to 875 flowsheet data entries within a 12-hour shift, equating to approximately 1 data entry per minute [6,7].

There is increased awareness among national government entities and professional health care organizations across the United States regarding the need to implement initiatives that address EHR documentation burden. For instance, the 21st Century Cures Act identified the following three goals for reducing clinician burden: (1) reduce the time and effort needed to document health information, (2) reduce the time and effort needed to meet regulatory requirements, and (3) improve usability [8]. Similarly, in 2021, the AMIA Task Force aimed to reduce clinician documentation burden by 25% within 5 years [5,9]. Additionally, the American Nursing Association’s Principles for Nursing Documentation recommend that nursing data entries should be meaningful and nonredundant [10]. Further, a prior study found that flowsheet redesign saved an average of 10 minutes per shift in flowsheets [11]. Other interventions, such as EHR optimizations and training, could improve clinician satisfaction, and nurses show increasing utilization of documentation efficiency tools once such tools are available [12,13].

The American Nursing Informatics Association (ANIA) developed the Six Domains of Documentation Burden conceptual framework, defining the factors that contribute to documentation burden as follows: reimbursement, regulatory, quality, usability, interoperability/standards, and self-imposed [14]. Nursing flowsheet documentation represents a significant amount of the overall documentation time for nurses, making it a prime area for burden evaluation through ANIA’s framework.

**Figure 1.**
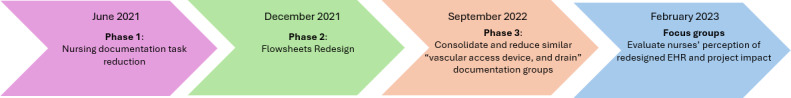
Improvement of nursing documentation experience: phased implementation and focus groups. EHR: electronic health record.

During the COVID-19 pandemic, our academic health system (AHS) reduced nursing flowsheet documentation by requiring only the documentation of critical assessments. Along with national calls to action for reducing documentation burden, this streamlined documentation approach served as a catalyst for the chief nursing officer, IT analysts, and nursing informatics team to optimize the nursing digital experience across the enterprise. We adopted a phased implementation approach to address challenges. In 2021, nursing informatics and IT analysts led nursing documentation enhancement workgroups with direct care nurses across the AHS. Nurses highlighted areas of the EHR system that were burdensome and suggested improvements. The nursing informatics team analyzed data from the EHR to identify flowsheet rows with minimal to low usage rates and brought these up as discussion points during workgroup meetings. Additionally, nursing informatics and IT analysts conducted an analysis of the various “vascular access device and drain” documentation groups. Cross-referencing these documentation groups revealed opportunities to consolidate similar documentation groups. During the workgroup sessions, direct care nurses expressed a preference to group and reduce the amount of “vascular access device and drain” documentation groups. Nursing informatics and IT analysts presented proposed changes to the steering council (comprised of the chief nursing officer and cross-campus nursing senior directors) for approval. Projects for implementation were prioritized into the following three phases (Figure 1): phase 1 focused on reducing nonmeaningful nursing documentation tasks, phase 2 involved redesigning flowsheets, and phase 3 involved consolidating and reducing similar “vascular access device and drain” documentation groups. Throughout each phase of the implementation, the nurse workgroup participants contributed recommendations and served as liaisons, gathering feedback from respective units and specialties. After the implementation of the three phases, our AHS sought to evaluate the project’s impact and determine if further improvements were needed.

### Objective

We aimed to assess nurses’ perceptions of the redesigned EHR, evaluate the impact of documentation interventions, and identify future improvement needs.

## Methods

### Setting

Our assessment, which used qualitative and quantitative methods, was conducted at an AHS with 4 teaching hospitals in the northeastern region of the United States; each teaching hospital was designated as an American Nurses Credentialing Center Magnet site. The AHS employs almost 10,000 nurses, and it implemented the current EHR system in 2012.

### Design

In our multimethod assessment, qualitative data were collected during 5 focus group sessions. A focus group method was chosen to evaluate the phased interventions and participants’ lived experiences with documenting in the EHR [15]. Focus groups were selected for their ability to facilitate active interaction among participants and generate opinions, suggestions, and feedback through group dynamics [16]. Our quantitative data consisted of monthly data supplied by the EHR vendor, which summarized the time nurses spent in the EHR system. The system logged the time each user spent within the EHR by tracking the time spent performing clicks, scrolls, keystrokes, and mouse movements [17]. These quantitative data could be divided based on EHR-related activities, such as nursing flowsheet documentation, medication administration, and task management.

### Sample

The quality improvement project team recruited inpatient registered nurses across the AHS through an electronic flyer. Recruitment was performed during a pre-existing focus group process known as “Ideas for Innovation in Nursing.” This process provided an opportunity for nurses to share their ideas about a given topic (ie, use of 3D printing in nursing practice). The full-page electronic flyer was embedded in the AHS’s monthly nursing science newsletter, which has a distribution list of approximately 8000 nurses and provides information about upcoming focus groups. The registered nurses who were interested in participating in the focus groups were required to complete the electronic registration form, which involved selecting a preferred session date and time.

### Data Collection and Analysis

The quality improvement team members (DJ, JW, DD, BD, KEZ, and KO) met to develop open-ended questions for guiding focus group discussions. Prior to conducting the focus groups, DJ, JW, and DD were trained by BD and KEZ, who were experienced in qualitative methods and focus group facilitation. Additionally, BD and KEZ participated as comoderators in each focus group discussion. The moderator (DJ) led the focus group sessions, introduced the purpose and formatting of the focus group, and facilitated the questions. The observers (JW, DD, BD, and KEZ) used a template to document notes on and observations of participants’ sentiments, behaviors, and nonverbal reactions. Sessions were conducted via a web conferencing platform. The focus group sessions ranged from 60 to 90 minutes in duration.

At the end of each focus group, participants received a link to an anonymous survey. The survey, which was administered via an administration platform, gathered demographic information, self-assessments of EHR competency, and feedback specifically about the focus group sessions. Participants’ perceived level of EHR competency was defined by using Benner’s [18] novice to expert theory. Benner’s [18] model was initially used to understand how nurses develop clinical competence, but it has expanded to evaluate EHR skills [19]. The project team members (DJ, JW, DD, BD, KEZ, and KO) debriefed at the end of each session to review notes, identify themes, and compare findings from prior focus group discussions. Sessions were recorded and transcribed by using a web videoconferencing platform. The transcriptions were validated by the project team. The project team members (DJ, JW, DD, BD, KEZ, and KO) met to confirm that saturation was reached. Transcriptions were entered into ATLAS.ti Web (version v9.4.3; ATLAS.ti Scientific Software Development GmbH)—a qualitative data analysis software for coding. Thematic analysis with an inductive coding process was used to discover themes. The primary coder (DJ) completed initial coding and developed the codebook. The secondary coder (LG) independently reviewed and validated the codes. The coders met to identify patterns and themes within the data, leveraging The Six Domains of Burden conceptual framework to organize the codes and examine the multifaceted burden experienced by nurses [10]. All quotes were reviewed by DJ and LG to reach consensus on discrepancies and further refine codes.

EHR activity data regarding the time spent in flowsheets were calculated for February 2023—the same month as when the focus group discussions were conducted. These activity data were time-stamped, allowing for the calculation of the time spent specifically within that month. The average time spent in a documentation activity was calculated as a percentage for all nurses in the AHS system. This quantitative approach was designed to provide context to the qualitative data. This quality improvement project was reported in accordance with the SQUIRE (Standards for Quality Improvement Reporting Excellence) 2.0 guidelines [20].

### Ethical Considerations

This project was undertaken as part of a quality improvement project for evaluating nursing documentation experiences with the EHR. The project team completed an NYU Langone Health Institutional Review Board–approved quality improvement self-certification. Participants voluntarily registered to take part in the focus group discussions and were not provided with any form of compensation. At the beginning of each focus group, participants provided verbal agreement for sessions to be recorded and transcribed while maintaining their anonymity.

This project was determined to not meet the criteria for human subjects research, as guided by the NYU Grossman School of Medicine’s Institutional Review Board policy.

## Results

### Participants

A total of 50 nurses responded via the electronic flyer’s registration link. Of those, 20 participated in the focus groups, with an 85% (n=17) response rate for the demographic survey; 3 participants declined to complete the survey. Each focus group composition was made up of nurses from different hospitals and units. The focus group participants’ demographic characteristics are listed in Table 1. Of the 17 respondents, most (n=12, 71%) were full-time employees, and the participants’ primary area of practice was inpatient units (n=10, 59%). Further, 35% (n=6) of participants had been working at the AHS for 3 to 5 years, 47% (n=8) had 3 to 5 years of EHR system experience, and 53% (n=9) self-reported being competent in the EHR system.

**Table 1.** Focus group participants’ demographics (n=17).

Demographics	Participants, n (%)
Employment status	
Full-time	12 (71)
Part-time	4 (24)
Per diem	1 (6)
Area of practice	
Inpatient (acute, intensive care unit, maternal/child)	10 (59)
Emergency medicine	1 (6)
Perioperative	4 (24)
Other	2 (12)
Years at academic health system	
1‐2	2 (12)
3‐5	6 (35)
6‐10	4 (24)
11‐15	2 (12)
16‐20	1 (6)
>20	2 (12)
EHR^a^ system experience (years)	
1‐2	2 (12)
3‐5	8 (47)
6‐10	5 (29)
>10	2 (12)
EHR system proficiency	
Advanced beginner	1 (6)
Competent	9 (53)
Proficient	5 (29)
Expert	2 (12)

a EHR: electronic health record.

### Focus Group Findings

#### Overview

Herein, our findings are presented over the following six major themes: positive feedback, usability and workflow opportunities, nuisance, training and education, communication, and time spent in the system. Quantitative analysis results for EHR activity data regarding the time spent in flowsheets are reported for the “time spent in the system” theme, which included participants’ subjective descriptions of how their shift time is spent; details of the analysis are presented in the *Time Spent in the EHR System* section.

#### Positive Feedback

##### Overview of Positive Feedback

Seventeen nurses in 5 focus groups provided positive feedback on the benefits of improved EHR workflows, including nursing documentation task management, flowsheet documentation, communication, and device and vendor integration. Positive feedback included the following subthemes: safe patient care, efficiency, and ease of use. Nurses reported positive sentiments on the nursing documentation programs that were implemented to improve documentation, such as the following:

*The central lines....The date change row, all that I appreciate, because we did not have that for a while, and a lot will get lost in translation....There is a lot of improvements especially with skin,...Central lines catheters and drains. I appreciate all the changes that have been done, it’s easier to just go back and backtrack to see when the last dressing was done or how it looked before the wound images.*

[Participant 15]

##### Safe Patient Care

Five nurses in 3 focus groups reported that the EHR system supported safe patient care delivery. Two nurses commented on the ease of viewing patients’ surgical history along the continuum of care. One participant said:

*I think it’s a great system. You know, coming from the paper charting to this...when you think about it. How crazy that time was - I cannot imagine not having the electronic chart....Really, it’s great. It’s great for follow up. It’s great for care. I think it improves health.*

[Participant #1]

##### Efficiency

Nurses commented that efficiency tools, such as the vital signs integration and copy and paste, aid in reducing manual documentation, resulting in less time spent in the EHR system. For instance, a participant stated:

*I find it helpful when you hook them up to the monitor and the vitals automatically get transferred....Very helpful for us because we see so many patients a day....It saves us the time of having to sit there manually inputting them.*

[Participant #18]

Focus group participant #1 also reported that “the more we can integrate the better.”

##### Ease of Use

Four nurses appreciated the EHR task management feature and noted that the enhancements made the workflow easier. Six nurses expressed that the flowsheets were intuitive for documentation and were streamlined. One said:

*It’s super-duper easy. I usually take 5 minutes to finish my baseline charting.*

[Participant #13]

##### Interdisciplinary Communication

An EHR functionality allowing secure, direct messaging between clinicians was cited by 4 nurses in 3 different focus groups as something that improved their clinician experience through convenience and features such as the ability to send an image of an electrocardiogram directly to a covering radiology cardiology resident. The direct messaging feature was appreciated, while workflows involving calls to unit-based landline phones were highlighted as being particularly disruptive. The nurses carried work-issued mobile devices, which can be called directly. Further, a participant stated:

*I mean, I love this system. Because whatever I do, we connect with each other.*

[Participant #13]

### Usability and Workflow Opportunities

Fourteen nurses in 5 focus groups reported usability and workflow opportunities in the EHR. Nurses commented on the desire for the standardization and consistency of documentation template design. For example:

*There are some things that go in alphabetical order. And then something else will go in order of head to toe. And then something else will go in order of like, abnormal, abnormal, abnormal, and then normal. And so, it feels like it changes...if it were just consistent, I think it would be easier.*

[Participant #9]

Three nurses commented on the need for specialty-specific documentation templates to support clinical workflows. One reported:

*I think the flowsheets are more catered to inpatient nurses...for telehealth nursing we use the flowsheets that were already in [redacted] but I think most of the questions in there are not telehealth specific, and also the nursing assessment is completely different over the phone because we obviously can’t visualize the patient. I think there’s definitely a lot of improvement* that *we can go forward with documentation in terms of telehealth.*

[Participant #3]

### Nuisance

#### Redundant Documentation

Four nurses in 2 different focus groups described redundant documentation during the admission process. The EHR frequently prompted them for information that was elsewhere in a patient’s chart:

*It asks me to put in if the patient received their COVID vaccination....I have to click on it but it’s already on the storyboard. So, I think that’s just added documentation that is unnecessary on our end.*

[Participant #6]

Eight nurses reported that in some cases, documentation was repetitive, as they had to document the same finding in several different places within the EHR. Examples included multiple places for documenting paper tape, skin assessments, patient activity, and patient positioning. Nurses described how the repetition was time-consuming and that they desired documentation to be streamlined.

#### Nonmeaningful Documentation

Four nurses in 2 focus groups described nuisances related to nonmeaningful documentation. They cited oral assessment items that needed to be documented for all patients, irrespective of patients’ needs; verbalized emotional states; subjective findings; and national standards, which necessitate documenting every 15 minutes. Although nurses understood the nature of hospital protocols, they felt that some documentation was more for “covering” oneself rather than serving an actual clinical purpose.

### Training and Education

Ten nurses in 4 focus groups discussed training and education related to the EHR. Four nurses described the learning curve for new nurses to acclimate to the EHR system. One participant said:

*I also like the tip sheets because if you don’t do something for quite a long time, you forget how to do it. The tip sheets are very helpful - tell you how to document.*

[Participant #4]

Two nurses reported their dissatisfaction with the EHR optimizations and communication method. For example:

I think it’s very easy to miss those general broadcast emails. I think just like, batching changes would probably be most helpful. This group of changes is happening rather than 1 change here or 1 change here and there’s 10 different emails about it.

[Participant #8]

### Communication

Six nurses discussed how the EHR system supports communication. Three nurses commented on the clinical mobile device and its strengths and weaknesses particularly around meeting patients’ needs regarding their preferred language. The clinical mobile device, which paired with a mobile app for interpreter support, excelled at simplifying the process of connecting to a remote interpreter:

*The steps saved from when you used to call, and they ask you what department you were calling from and what language you needed. That saves you a few minutes and that is priceless on its own.*

[Participant #10]

### Time Spent in the EHR System

Participants self-reported that 10% to 50% of their shift is spent documenting in the EHR system, and many perceived this time to be appropriate. The EHR vendor time data were analyzed during the focus group period. The EHR vendor time data for February 2023 revealed that the average EHR time spent in flowsheets was 31.11% per 12-hour shift. In relation to the time spent in flowsheets, one participant stated:

*I would definitely say the shift assessment takes up your biggest amount of time. You want to be thorough; you don’t want to miss things. So, that really is the largest amount of time.*

[Participant #1]

## Discussion

### Principal Findings

The project’s aims were to explore nursing documentation experiences related to the EHR and evaluate how the documentation reduction interventions impacted perceptions. Themes included positive feedback, usability and workflow opportunities, nuisance, training and education, communication, and time spent in the system. Nurses perceived that the EHR supported the delivery of safe patient care and care team communication. Participants complimented the EHR system’s easier flow and remarked on the general improvements. The project revealed that while the nurses overall had a positive experience with using the EHR system, there are further opportunities to optimize the EHR design. The implementation of voice recognition tools for nursing documentation supports the capture of patient assessments in real time by reducing the average time spent documenting by more than 2.6 minutes per assessment [21].

The focus group participants did not describe burden from using the EHR; rather, they noted redundant and nonmeaningful documentation as a nuisance. Focus group participants suggested solutions for reducing nonmeaningful documentation, such as optimizing the oral health assessment tool and only requiring oral health assessment documentation for ventilated and tracheostomy patients, which aligns with the AHS’s policies and standards. Due to this project, this enhancement request was implemented at the AHS, with positive sentiments from nurses. Many documentation burden reduction interventions have shown improved satisfaction with the EHR among clinicians [22].

The Six Domains of Documentation Burden conceptual framework on EHR documentation burden indicates that most health care system cultures adhere to the ideology of “if it’s not documented, it’s not done” [10]. As a result, some nurses may document due to perceived legal implications. Focus group participants discussed documentation volume and the sense that they document too much. Health care system accreditation organizations have recognized the need to reduce documentation burden. In 2023, The Joint Commission aimed to eliminate 14% of standards and updated 13 standards [23]. Individual hospitals can make impacts to address EHR documentation barriers and reduce documentation through shared governance workgroups that include frontline nurses [24].

The project’s findings reveal opportunities for continuous EHR education. Per the focus group participant survey results, 47% (8/17) of participants had 3 to 5 years of EHR experience, and 53% (9/17) of participants self-reported being competent in the system. Some focus group participants discussed not being familiar with efficiency tools, such as the EHR search toolbar for quickly finding information within a patient’s chart. Training sessions can enhance perceptions of efficiency [25]. Future studies should explore the use of the Digital Literacy, Usability, and Acceptability of Technology Instrument for Healthcare—a validated instrument for evaluating frontline nurse competency and usability with respect to the implementation of continuous health IT learning programs [26].

The findings from the focus group discussions prompted the project team to implement strategies that aimed to augment the nursing documentation experience in the EHR system. To support continuous EHR learning, the nursing informatics and IT training teams provided nursing staff with interactive enrichment classes that focused on nursing efficiency and common EHR workflows. The training content was developed based on frontline staff recommendations. The nursing informatics and IT training teams conducted nursing wellness fairs as drop-in opportunities during shifts to showcase EHR efficiency and tips. Remote sessions were offered for nurses to learn about upcoming documentation enhancements that would improve workflow and to provide feedback. As a result of the focus groups, the oral health assessment was optimized such that it only displayed in the EHR for ventilated and tracheostomy patients, rather than being required for all patients, as part of a shift documentation assessment; this change aligned with the AHS’s policies and standards. Additionally, efforts were made, in collaboration with direct care nurses, to streamline and reduce wound and skin documentation.

We acknowledge limitations related to our qualitative and quantitative approaches. Our sample did not include nurses from pediatric, behavioral health, or rehabilitation units. The recruitment flyer was distributed within an email newsletter and may not have been seen by all nurses. Further, due to this being a quality improvement project, we could not look at individuals’ utilization patterns, and quantitative metrics were summarized for all inpatient nurses at the hospital. Additionally, perceived documentation time spent in the EHR system was self-reported by focus group participants, and the EHR vendor time data analysis was not limited to focus group participants. Moreover, the focus group discussions were not limited to nurses who were employed prior to the documentation reduction interventions. However, this group made up a small fraction of the interviewees. Lastly, self-reported time spent documenting in the EHR might be influenced by group conformity bias. Participants in focus groups may be hesitant to express views that dissent from those of the group. Future work should explore a validated method for measuring burden [27].

### Conclusion

Our focus group discussion findings suggest that the implemented nursing documentation improvement interventions had an overall positive impact on the nurses’ EHR experience. As health care technology and documentation requirements continue to advance, the EHR experience requires ongoing evaluation. Analyzing frontline nursing perspectives in the restructuring of EHR workflows is imperative for identifying interventions that support nurses’ satisfaction with the EHR. Future work is needed in supporting nurses after the EHR system onboarding training period.

## Abbreviations

AHS: academic health system
AMIA: American Medical Informatics Association
ANIA: American Nursing Informatics Association
EHR: electronic health record
HITECH: Health Information Technology for Economic and Clinical Health
SQUIRE: Standards for Quality Improvement Reporting Excellence
